# The use of video laryngoscopy outside the operating room: A systematic review

**DOI:** 10.1371/journal.pone.0276420

**Published:** 2022-10-20

**Authors:** Emma J. Perkins, Jonathan L. Begley, Fiona M. Brewster, Nathan D. Hanegbi, Arun A. Ilancheran, David J. Brewster

**Affiliations:** 1 Alfred Health, Melbourne, VIC, Australia; 2 Intensive Care Unit, Cabrini Hospital, Malvern, VIC, Australia; 3 Department of Anaesthesia, Royal Women’s Hospital, Parkville, VIC, Australia; 4 Central Clinical School, Monash University, Melbourne, VIC, Australia; Azienda Ospedaliero Universitaria Careggi, ITALY

## Abstract

This study aimed to describe how video laryngoscopy is used outside the operating room within the hospital setting. Specifically, we aimed to summarise the evidence for the use of video laryngoscopy outside the operating room, and detail how it appears in current clinical practice guidelines. A literature search was conducted across two databases (MEDLINE and Embase), and all articles underwent screening for relevance to our aims and pre-determined exclusion criteria. Our results include 14 clinical practice guidelines, 12 interventional studies, 38 observational studies. Our results show that video laryngoscopy is likely to improve glottic view and decrease the incidence of oesophageal intubations; however, it remains unclear as to how this contributes to first-pass success, overall intubation success and clinical outcomes such as mortality outside the operating room. Furthermore, our results indicate that the appearance of video laryngoscopy in clinical practice guidelines has increased in recent years, and particularly through the COVID-19 pandemic. Current COVID-19 airway management guidelines unanimously introduce video laryngoscopy as a first-line (rather than rescue) device.

## Introduction

Tracheal intubation occurring outside the operating room (OR) typically involves a critically unwell patient. These intubations occur predominantly in the emergency department (ED) or intensive care unit (ICU), but may also involve a deteriorating patient on a hospital ward. Intubation outside the OR presents greater difficulty to the airway, with a significantly increased incidence of adverse events and risks to patient safety [1, 2]. Reasons for this include availability of skilled staff in an emergency, case mix and working environment [3]. In the recent INTUBE study, 45.2% of intubations outside the OR experienced at least 1 major adverse peri-intubation event and over 3% were complicated by cardiac arrest [2].

First-pass intubation success is particularly important in the critically ill and is associated with improved hospital survival in this group [2]. As the number of intubation attempts increases, so too does the incidence of adverse events and hospital mortality [2, 4]. As such, every effort should be made to maximise first-pass intubation success outside the OR. Strategies to increase first-pass success include choice of an experienced clinician (such as a consultant anaesthetist), positioning, adequate muscle relaxation, and use of equipment [5–7]. Specifically, airway operators must consider whether to use direct laryngoscopy (DL) or video laryngoscopy (VL). VL displays the glottis on an external monitor via a camera attached to the device blade, without requiring alignment of the oral-pharyngeal-tracheal axes. Furthermore, because the glottis is displayed on an external monitor, VL allows supervising clinicians real-time view, allowing them to provide tailored guidance to trainees [8, 9]. Historically, VL is often referred to in difficult airway management algorithms as a powerful rescue tool to be employed when initial intubation attempts are unsuccessful [10, 11]. Recently, the use of VL has increased, which is likely due to multiple factors including the improved glottic view that VL offers compared to DL, and its increased availability and affordability [12, 13]. There has also been an increase in the uptake of VL during the COVID-19 pandemic, with many airway management guidelines now recommending VL as a first line (rather than rescue) device [6, 7, 13]. Within the OR, it has been found that VL may reduce the number of failed intubations, particularly among patients presenting with a difficult airway [14]. However, there has been conflicting results of early VL studies outside OR. There is no current consensus on the use of VL outside the OR; specifically, whether it should be used ahead of DL, whether it is best used by trainees, consultants or both, and what benefit to patient outcomes it may offer. Existing systematic reviews on the use of VL were conducted prior to the COVID-19 pandemic, and do not evaluate the most recent evidence on the use of VL outside the OR.

This study was designed to conduct an up-to-date review of the existing literature on how VL is used outside the OR. Specifically, it aims to (1) search for and summarise the recent evidence for the use of VL outside the OR and (2) describe how VL appears in current clinical practice guidelines for airway management outside the OR.

## Materials and methods

A structured literature search adhered to the Preferred Reporting Items for Systematic Reviews and Meta-analysis (PRISMA) recommendations and was registered with PROSPERO [15]. A search was conducted across two databases (MEDLINE and Embase) on September 8, 2021, looking at the use of VL outside the OR. Keywords searched were “laryngoscopy” + “video recording”, “video laryngoscopy”, “video assisted laryngoscopy”, “penta airway”, “king vision” or “mcgrath mac”, in conjunction with “intensive care units”, “critical illness”, “ICU(s)”, “emergency department”, “emergency service/hospital” or “critically ill”. Pre-defined exclusion criteria were studies not in English, not in ED/ICU/ward based/critically ill settings, pre-hospital settings, simulation studies, involving students, conference abstracts only, commentary/editorials or neonatal/paediatric papers. Furthermore, articles were limited to those published between 1 January 2011–8 September 2021.

**Fig 1:**
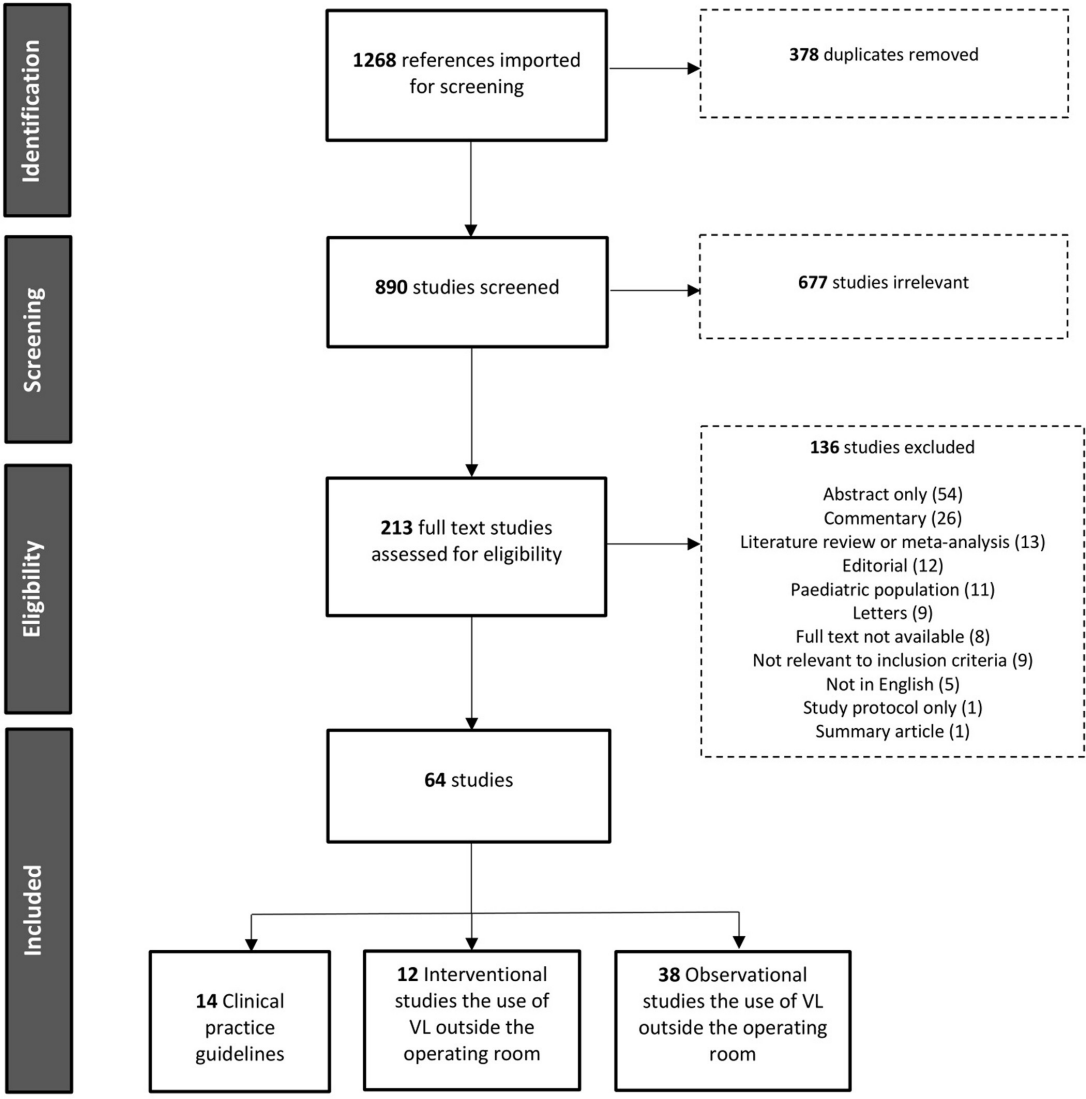
PRISMA flow diagram of studies identified, screened and included in this review.

All resulting references underwent title and abstract screening using Covidence software (Veritas Health Innovation Ltd, Melbourne, Victoria). Articles were initially screened by a single author, and any articles that were not clearly able to be included or excluded were then discussed amongst the team of authors. Full texts were then extracted and screened by two authors for relevance to pre-defined inclusion and exclusion criteria (Fig 1).

Both clinical practice guidelines and consensus statements (for both intubation and advanced life support/cardiopulmonary resuscitation) were included. This yielded both primary and secondary research papers discussing the use of VL outside the OR (ED, ICU and ward-based setting). As this is a review article, we limited citations of other review articles or meta-analyses to the introduction and discussion and only primary data was used in our descriptive analysis.

Clinical practice guidelines and consensus statements were identified and tabulated (Table 1). Articles discussing the use of VL outside the OR were identified and tabulated separately, with Table 2 detailing interventional studies and Table 3 detailing observational studies. Descriptive analysis was performed to assess how VL is used outside the OR, what the evidence is for its use outside the OR and the appearance of VL in current clinical practice guidelines. The quality of included papers was assessed using the Critical Appraisal Skills Programme (CASP 2018) and the Medical Education Research Study Quality Instrument (MERSQI) [16, 17].

**Table 1:** Clinical practice guidelines and consensus statements recommending VL outside the OR.

	Author (Year)	Country of publication	Society/Expert Group	Methodology for guideline development	Patient group	Type of VL	Recommendations	Evidence base
**1**	**Apfelbaum et al. (2013)** [18]	United States	American Society of Anesthesiologists	Literature search + expert consensus	Difficult airway	Not specified	This guideline prompts the airway operator to consider VL as an initial approach to intubation based on “relative merits and feasibility”. This guideline also lists VL as an alternative approach to difficult intubation, when the first attempt at intubation has been unsuccessful.	Combination of category I–category IV evidence
**2**	**Barati et al. (2020)** [19]	Iran	Iranian Heart Association	Not specified	Cardiopulmonary resuscitation	Not specified	This guideline recommends that intubation performed during CPR should be done with the help of VL if possible.	Not specified
**3**	**Brewster et al. (2020)** [6]	Australia and New Zealand	Safe Airway Society	Expert consensus	Adults with COVID-19	Macintosh video laryngoscope Hyperangulated video laryngoscope	Recommends VL as the device of choice for first attempt at intubation (when operator is proficient in its use)	Category IV—Consensus statement
**4**	**Cook et al. (2020)** [7]	United Kingdom	Difficult Airway Society, the Association of Anaesthetists the Intensive Care Society, the Faculty of Intensive Care Medicine and the Royal College of Anaesthetists	Expert consensus	Critically ill adults with COVID-19	Not specified	Recommends that laryngoscopy should be undertaken with the device that is most likely to achieve prompt first-pass success. In most fully trained airway managers this is likely to be a videolaryngoscope.	Category IV—Consensus statement
**5**	**Frerk et al. (2015)** [20]	United Kingdom	Difficult airway society	Literature search + expert consensus	Unanticipated difficult airway	Not specified	Does not specifically recommend VL within the guideline, but comments on the fact that the role of VL in difficult intubation is recognised, and all anaesthetists should be skilled in its use.	Combination of category I–category IV evidence. Individual techniques have not been listed against their level of evidence.
**6**	**Higgs et al. (2018)** [21]	United Kingdom	Difficult Airway Society (DAS), Intensive Care Society (ICS), Faculty of Intensive Care Medicine (FICM), and Royal College of Anaesthetists (RCoA)	Literature search + expert consensus	Critically ill adults	Macintosh video laryngoscope is suggested when VL is used as a first line device. Hyperangulated video laryngoscope suggested when VL is used as a rescue device.	Recommends the early use of VL. Recommends that VL should be available and considered as an option for all intubations of critically ill patients. If difficult laryngoscopy is predicted in a critically ill patient, VL should be actively considered from the outset.	The quality of evidence for these recommendations varied considerably (GRADE level 2+ to 5) and in its absence, consensus was sought. Published data on VL in critically ill patients are generally poor quality, with limited evidence from ICU and ED populations.
**7**	**Myatra et al. (2017)** [13]	India	All India Difficult Airway Association	Literature search + expert consensus	Critically ill	Not specified	Mentions VL as an option to consider for the initial intubation attempt.	Developed based on available evidence but where this is lacking, recommendations are based on consensus opinion of airway experts
**8**	**Nasa et al. (2021)** [22]	India	Critical care physicians actively involved in the management of patients with COVID-19 acute respiratory failure	Delphi method	COVID-19 related acute respiratory failure	Not specified	Expert consensus suggests consideration of VL during tracheal intubation in context of COVID-19 related acute respiratory failure	Category IV—Consensus statement
**9**	**Nolan et al. (2020)** [23]	Not specified	European Resuscitation Council	Not specified	COVID-19 patients requiring cardiopulmonary resuscitation	Not specified	For in-hospital cardiac arrests, this guidelines suggests that airway operators should consider VL if provider is familiar with its use	Not specified
**10**	**Oh et al. (2021)** [24]	Korea	Not specified	Not specified	Adults requiring cardiopulmonary resuscitation	Not specified	Recommends that during CPR, VL should be considered for intubation.	Not specified
**11**	**Piepho et al. (2015)** [10]	Germany	German Society of Anesthesiology and Intensive Care	Expert consensus	Difficult airway	Macintosh video laryngoscope Hyperangulated video laryngoscope	This guideline recommends that for an unexpected difficult airway, VL may be used as an “alternative strategy”.	Category IV–Expert consensus
**12**	**Quintard et al. (2019)** [25]	France	French Society of Anaesthesia and Intensive Care Medicine (SFAR) and French-speaking Intensive Care Society (SRLF)	GRADE method	Critically ill	Not specified	This guideline recommends the use of VL as an initial option for intubation when MACOCHA score ≥3, or as for second-attempt intubation when MACOCHA score <3.	Category IV—Consensus statement
**13**	**Sharma et al. (2020)** [26]	United States	Society of Vascular & Interventional Neurology (SVIN), Society of NeuroInterventional Surgery (SNIS), Neurocritical Care Society (NCS), European Society of Minimally Invasive Neurological Therapy (ESMINT) and American Association of Neurological Surgeons (AANS) and Congress of Neurological Surgeons (CNS) Cerebrovascular Section	Not specified	COVID-19 patients requiring emergency endovascular treatment for ischaemic stroke	Not specified	Recommends that VL should be used for patent requiring urgent endovascular treatment for ischaemic stroke during COVID-19 pandemic.	Category IV—Consensus statement
**14**	**Singh et al. (2020)** [27]	India	Indian Resuscitation Council (IRC)	Not specified	COVID-19 patients requiring cardiopulmonary life support	Not specified	Recommends that in cardiopulmonary resuscitation of COVID-19 patient, VL should be used if the airway operator is familiar with its use.	Literature search + expert consensus

**Table 2:** Interventional studies on the use of VL outside the OR.

	Author (Year)	Country	Setting	Patient group	Airway operator(s)	Study design	Type of VL	Outcome(s)
**1**	**De Jong et al. (2013)** [28]	France	ICU	Critically ill	Not specified	Interventional before and after study	Combo videolaryngoscope	Incidence of difficult laryngoscopy and/or difficult intubation (VL < DL, p = 0.01) Severe, life threatening complications (no difference)
**2**	**Driver et al. (2016)** [29]	United States	ED	Adult patients in resuscitation bays of ED who were to undergo emergency orotracheal intubation using DL as device choice for first attempt.	Emergency medicine trainees Emergency medicine physicians	RCT	C-MAC	First-pass success (no statistically significant difference between VL and DL) Duration of intubation attempt (no difference between DL and VL) Aspiration pneumonia (no difference between DL and VL) Hospital length of stay (no difference between DL and VL)
**3**	**Gao et al. (2018)** [30]	China	ICU	Critically ill	ICU physicians	Randomised non-blinded trial	Med. Adult type Video Laryngoscope VL300M, Zhejiang UE Medical Corp	First-pass success (no statistically significant difference between DL and VL)
**4**	**Griesdale et al. (2012)** [31]	Canada	Not specified	Critically ill	Non-anaesthesiology residents or medical students	Pilot randomised trial	GlideScope	Cormark-Lehane grade 1 glottic view (VL 85%, DL 30%, p<0.001) Clinical outcomes (no difference between DL and VL)
**5**	**Groombridge et al. (2021)** [32]	Australia	ED	All ED intubations	ED consultants, ED registrars, anaesthetic consultants, anaesthetic registrars, ICU consultants, ICU registrars	Interventional study	Storz C-MAC	VL was more likely to be used for ED intubations during the COVID-19 pandemic compared to prior to the COVID-19 pandemic.
**6**	**Ilbagi et al. (2021)** [33]	Iran	ED	Al patients undergoing intubation in ED	Novice physicians	Randomised trial	GlideScope	Hemodynamic changes (VL > VL, p< 0.001)
**7**	**Janz et al. (2016)** [34]	United States	ICU	Critically ill	Not specified	Randomised, parallel-group pragmatic trial	McGrath Video Laryngoscope GlideScope	First-pass success (VL 68.9% vs DL 65.8%; *p* = 0.68). Glottic view (VL > DL). Time to intubation, lowest arterial oxygen saturation, complications and in-hospital mortality (no difference between VL and DL)
**8**	**Kim et al. (2016)** [35]	Korea	ED	Patients undergoing CPR	Experienced operators	Prospective randomised controlled study	GlideScope	Intubation success during CPR (no significant difference between VL and DL). Speed of intubation during CPR (no significant difference between VL and DL). Sakles Complications of intubation during CPR (no significant difference between VL and DL). Completion of intubation without interruption of chest compressions (VL > DL).
**9**	**Lakticova et al. (2015)** [36]	United States	ICU	Critically ill	Not specified	Controlled non-randomised trial	GlideScope	Oesophageal intubations (VL (0.4%) < DL (19%)). Difficult intubation rate (VL (7%) < DL (22%).
**10**	**Lascarrou et al. (2017)** [37]	United States	ICU	Critically ill	Not specified	Randomised clinical trial	McGrath MAC	First-pass success (no statistically significant difference between VL and DL). Severe life-threatening complications (VL 9.5% vs DL 2.8%, p = 0.01).
**11**	**Silverberg et al. (2015)** [38]	United States	ICU	Critically ill	Pulmonary and critical care medicine fellows	Randomised controlled trial	GlideScope	First-pass success (VL (74%) > DL (40%), p < 0.001).
**12**	**Yeatts et al. (2013)** [39]	United States	ED	Trauma patients	Emergency medicine or anaesthesiology residents with a minimum of 1 year previous intubation experience.	Randomised controlled trial	GlideScope	Survival to hospital discharge (no significant difference between VL and DL). Time to intubation (VL > DL, p<0001). Incidence of low oxygen saturation (VL > DL, p = 0.004).

**Table 3:** Observational studies on the use of VL outside the OR.

	Author (Year)	Country	Setting	Patient group	Airway operator(s)	Study design	Type of VL	Outcome(s)
**1**	**Amalric et al. (2020)** [40]	France	ICU	Critically ill	Novice operators (1–5 previous experience with VL) vs expert operators (>15 previous experiences with VL)	Observational study	McGrath MAC	First pass success using VL (87% for expert operators and <50% for notice operators) Complications of intubation including severe hypoxemia (VL < DL, p<0.001).
**2**	**April et al. (2017)** [41]	United States	ED	All patients intubated in ED (71% trauma)	ED physicians	Observational study	Not specified	First pass success (VL 90.9% vs DL 73.0%)
**3**	**Aziz (2013)** [42]	United States	Pre-hospital ED	Trauma	Not specified	Not specified	Glidescope Airtraq WuScope AWS C-MAC Bullard Lightward	Glottic view (VL > DL). Use of VL is growing.
**4**	**Brewster et al. (2021)** [43]	Australia and New Zealand	ICU	COVID-19 patients	ICU directors	Observational study	Not specified	VL was used 94% of the time during the airway management of patients with COVID-19.
**5**	**Brown et al. (2010)** [44]	United States	ED	All adults requiring intubation in ED (74% medical, 26% trauma, 1% unknown)	Interns, residents and emergency physicians	Observational study	Karl Strorz Video Macintosh Laryngoscope	Glottic view (80% with DL and 93% with VL, p<0.0001)
**6**	**Brown et al. (2015)** [45]	United States	ED	ED patients requiring intubation (both medical and trauma)	ED physicians and trainees	Observational study	C-MAC V-MAC GlideScope Flexible fibre optics	First-pass success (increased 6% during the past decade and is highest when a C-MAC video laryngoscope is chosen as the first device)
**7**	**Brown et al. (2020)** [46]	United States	ED	ED patients requiring intubation	Not specified	Observational study	Hyperangulated VL Standard geometry VL	First-attempt success was significantly higher with all VL (90.9%, 95% CI = 88.7 to 93.1) versus all DL* (81.1%, 95% CI = 78.7 to 83.5) *DL in this study refers to DL augmented by laryngeal manipulation, ramped patient positioning and the use of a bougie compared to unaided VL
**8**	**Carlson et al. (2015)** [47]	United States	ED	Adults with gastrointestinal bleeding	Not	Observational study	Not specified	First pass success (no difference between VL and DL) Glottic view (no difference between VL and DL) Need to change device (no difference between VL and DL)
**9**	**Chan et al. (2021)** [48]	Singapore	ED	Majority medical indications	Majority postgraduate year 5 trainees, fellows and attending physicians	Observational study	Claurs video system C-MAC standard blade C-MAC D blade C-MAC straight blade McGrath video laryngoscope	First-pass success (no statistically significant difference between VL and DL) VL was the most commonly used device for emergency department intubations.
**10**	**Cho et al. (2015)** [49]	Korea	ED	Trauma	ED physicians (junior and senior) Physicians from other specialties (residents only)	Observational study	Multiple including: GlideScope Airtraq Truview	First pass success in difficult airway trauma patients (no statistically significant difference between VL and DL)
**11**	**Choi et al. (2015)** [50]	Korea	ED	Adults requiring intubation in ED (without cardiac arrest)	ED physicians (junior and senior) Physicians from other specialties	Observational study	GlideScope	First-pass intubation success (no statistically significant difference between VL and DL)
**12**	**Dodd et al. (2019)** [51]	United States	ED	Adults intubated in ED	Emergency physicians	Observational study	C-MAC	First-pass success (not significantly different when the screen was viewed (195/207; 94% [95%CI 91–97]) compared to when the screen was not viewed (284/301; 94% [95%CI 92–97]).
**13**	**Driver et al. (2020)** [52]	United States	ED	Patients aged over 14 intubated in ED	Emergency medicine postgraduate year 3 or 4, fellow, or attending physician	Observational study	Standard-Geometry VL (C-MAC Macintosh blades and the GlideScope Titanium Mac or disposable DirectView MAC blades) Hyperangualted VL (LoPro and GVL)	First pass success (no association between standard geometry VL and hyperangulated VL)
**14**	**Green et al. (2017)** [53]	Canada	ED ICU	Not specified	Emergency physicians ICU physicians	Observational study	Not specified	Most emergency physicians and ICU physicians use direct laryngoscopy with a Macintosh blade as a primary device.
**15**	**Hart & Goldstein (2020)** [54]	South Africa	ED	All patients requiring airway intervention in ED	Interns Medical officers Registrars Other (Medical students, paramedic students and trauma nurse trainees)	Observational study	GlideScope	First pass success (No difference between VL (81.7%) and DL (73.3%) (p-value 0.079)). Glottic view (VL > DL).
**16**	**Hawkins et al. (2021)** [55]	United States	All patients undergoing emergency intubation outside the operating room.	Patients with COVID-19.	Anaesthesiology residents, EM residents, anaesthesiologists, CRNA, emergency physicians and non-emergency physicians	Observational study	Not specified	VL was used significantly more in COVID-19 cases compared to non-COVID-19 cases
**17**	**Hypes et al. (2016)** [56]	United States	ICU	Critically ill	Pulmonary-critical care Critical care medicine Emergency medicine Internal medicine Family medicine Anaesthesia Surgery	Observational study	GlideScope C-MAC King Vision McGrath MAC	First-pass intubation success (achieved in 81.7% of patients intubated with VL, whereas >1 attempt was needed in 18.3%). Incidence on complications (greater when first-pass success not achieved)
**18**	**Hypes et al. (2017)** [57]	United States	ICU	Critically ill	Non-anesthetists	Observational study	GlideScope C-MAC King Vision McGrath MAC	First-pass success (VL (80.4%) > DL (65.4%, p < 0.001) Arterial oxygen desaturation (VL (18.3%) < DL (25.9%), p = 0.04) Oesophogeal intubation (VL (2.1%) < DL (6.6.%), p = 0.008)
**19**	**Joshi et al. (2017)** [58]	United States	ICU	Critically ill	Not specified	Observational study	GlideScope C-MAC Mcgrath MAC King Vision	First pass success using VL (reduced in the presence of blood in the airway, airway oedema, cervical immobility, and obesity).
**20**	**Kory et al. (2013)** [59]	United States	ICU Ward setting	Critically ill	Less experienced operators	Observational study	GlideScope	First-pass intubation success (VL (91%) > DL (68%), p<0.01). Rate of intubations requiring ≥3 attempts (VL (4%) < DL (20%), *p*< 0.01). Unintended esophageal intubations (VL (0%) < DL (14%), *p* < 0.01). Average number of attempts required for successful tracheal intubation (1.2 ± 0.56 for VL vs 1.7 ± 1.1 for DL, *p* < 0.01).
**21**	**Mallick et al. (2020)** [60]	India	ED	Patients presenting to ED requiring definitive airway management	Not specified	Observational study	KingVision	Mean time to intubate (no statistically significant difference between VL and DL). First-pass success (no statistically significant difference between VL and DL).
**22**	**Martin et al. (2020)** [61]	France	ICU	Critically ill	ICU physicians	Observational survey	MacGrath Airtraq GlideScope	The use of VL was reserved for difficult intubation in a majority of cases, rather than being used as a first line intubation device.
**23**	**Michailidou et al. (2015)** [62]	United States	ED	Trauma	Medical students, paramedics, PGY1-4 and attending)	Observational study	Not specified	Overall intubation success rate (VL 88% vs DL 83%, p = 0.05)
**24**	**Mosier et al. (2013)** [63]	United States	ICU	Critically ill	Residents, Pulm/CCM or CCM fellows, attending intensivists	Observational study	GlideScope C-MAC	First-pass success (VL > DL (78.6% vs 60.7%)). Ultimate success (VL > DL (98.3% vs 91.2%)). Oesophageal intubation (VL < DL (1.3% vs 12.5%)).
**25**	**Mosier et al. (2013)** [64]	United States	ED	Patients intubated with C-MAC or GlideScope VL in ED	Emergency medicine residents	Observational study	GlideScope C-MAC	First-pass success (No statistically significant difference between C-MAC VL and GlideScope VL).
**26**	**Noppens et al. (2012)** [65]	Germany	ICU	Critically ill patients with at least one predictor for difficult intubation	Junior physicians (<3 years clinical experience), senior physicians (>3 years clinic experience) and anaesthesiologists	Observational study	C-MAC	First-pass success (VL > DL, 79% vs 55%, p = 0.03). Glottic view (VL > DlL p<0.0001).
**27**	**Okamoto et al. (2018)** [66]	United States	ED	Cardiac arrest	Not specified	Observational study	C-MAC McGrath Airway Scope GlideScope	First attempt success (VL (78%) > DL (70%), p < 0.001). Glottic view (VL > DL, p < 0.001). Oesophageal intubation (VL < DL, p = 0.01).
**28**	**Weng et al. (2021)** [9]	Hong Kong	ED	Patients in emergency department requiring intubation	Attending and non-attending ED physicians	Observational study	GlideScope McGrath C-MAC	Overall first-pass success (VL < DL). First-pass success among non-attending emergency physicians (VL > DL). First-pass success among attending emergency physicians (VL < DL).
**29**	**Sakles et al. (2012)** [67]	United States	ED	All patients requiring intubation in ED	Not specified	Observational study	GlideScope	First-pass success (VL > DL, p = 0.03). Success rate when more than 1 attempt requires (DL > VL, p = 0.003). Overall intubation success rate (no statistically significant difference between VL and DL). Oesophageal intubation (VL < DL, p = 0.005).
**30**	**Sakles et al. (2014)** [68]	United States	ED	Patients with difficult airway characteristics	Emergency medicine PGY 1–3 and attenings	Observational study	GlideScope C-MAC	First-pass success in patients with difficult airway characteristics (VL > DL).
**31**	**Sakles et al. (2014)** [4]	United States	ED	Trauma	Not specified	Observational study	GlideScope	First pass-success using VL (75.6% in first year of study compared to 92.1% in seventh year of study, p = 0.008).
**32**	**Sakles et al. (2015)** [69]	United States	ED	Rescue intubations following a failed first attempt	Not specified	Observational study	C-MAC	Second-attempt intubation success (VL > DL). C-MAC is more successful than DL when used at second attempt intubation, regardless of device used in the first attempt.
**33**	**Sakles et al. (2017)** [70]	United States	ED	Non cardiac-arrest patients with predicted difficult airways requiring intubation in the ED	Not specified	Observational study	GlideScope C-MAC	First-pass success using VL in patients undergoing NMBA facilitated intubation (90%).
**34**	**Seisa et al. (2018)** [71]	United States	ICU	Critically ill	Critical care physicians	Observational study	Not specified	DL is a more common intubation device for intubating critically ill patients in ICU compared to VL.
**35**	**Silverberg & Kory (2014)** [72]	United States	Internal medicine / critical care training programs	Not specified	Critical care fellows	Observational study	Not specified	In critical care training programs, VL has become more available; however, it is still used uncommonly as a primary airway visualisation device.
**36**	**Smischney et al. (2019)** [73]	United States	ICU	Critically ill adults	Residents and fellows	Observational study	Not specified	The most common airway device used of VL (49%).
**37**	**Suzuki et al. (2018)** [74]	Japan	ED ICU	All video recorded tracheal intubations in ED and ICU during study period	First and second postgraduate years as non-expert operators and ≥third postgraduate year as experts.	Observational study	Pentax-Airway Scope King Vision McGrath MAC	First-pass success (Pentax and McGrath > King VL and Macintosh DL).
**38**	**Swaminathan et al. (2015)** [75]	United States	ED	Emergency medicine residents	N/a	Observational study	GlideScope Storz C-MAC McGrath King Vision Ambu-Pentax VividTrac	The majority of emergency residency programs train residents in the use of VL

## Results

### Search results

Following duplicate removal, our search generated 890 articles. Articles then underwent title and abstract screening and full text screening for relevance to our aims and exclusion criteria. This yielded a total of 64 articles to be included in our review. Guidelines not intended for use outside the OR, or guidelines that did not make mention of VL, were excluded. The 64 papers included 14 clinical practice guidelines or consensus statements (Table 1), 12 interventional studies including RCTs (Table 2) and 38 observational studies on the use of VL outside the OR (Table 3).

### Quality assessment

The quality of the included studies was primarily assessed used CASP tool [16]. Cross-sectional studies were assessed using the MERSQI tool [17]. Overall, the quality of included research was found to be low-moderate.

### Included airway guidelines

Guidelines were published from a range of countries as shown in Table 1. Table 1 also details data on society/expert group endorsement of the guidelines, methodology for guideline development, intended patient group, recommendations, and evidence base.

#### How is VL mentioned within guidelines?

Safe Airway Society guidelines for airway management of COVID-19 patients from Australia and New Zealand were the first to recommend VL as the first line device in COVID-19 patients when the airway operator is proficient in its use [6]. The Macintosh video laryngoscope and the hyperangulated video laryngoscope are the two types of VL referenced in this guideline. Guidelines from Cook et al. 2020, which come from consensus among Difficult Airway Society, the Association of Anaesthetists of the Intensive Care Society, and Faculty of Intensive Care Medicine and the Royal College of Anaesthetists, recommended that laryngoscopy be undertaken with the device that is most likely to achieve prompt first-pass success in patients with COVID-19 [7]. This guideline states that in most fully trained airway professionals, this is likely to be VL. Guidelines from Nasa et al. 2021, recommend considering VL for intubation of COVID-19 [22]. The guideline does not discuss a specific type of VL. Guidelines from Sharma et al. 2020, which are intended for use in COVID-19 patients requiring emergency endovascular treatment, state that VL should be used for intubation [26]. Guidelines from Nolan et al. 2020 and Singh et al. 2020 are specifically recommended for COVID-19 patients requiring cardiopulmonary resuscitation (CPR). Both of these guidelines state that VL should be used if the airway operator is familiar with its use [23, 27]. CPR guidelines from Barati et al. 2020 also recommend that intubation performed during CPR should be done with VL if possible [19]. Piepho et al. 2015 recommended that VL be used as an “alternative strategy” for management of an unexpectedly difficult airway [10]. All other included guidelines recommended early use of VL or the use of VL, rather than only as a rescue device.

### Non-guideline research papers on VL

Articles were published from a broad range of countries, with settings including one or multiple of ED, ICU and ward-based settings (see Tables 2 and 3). A majority of the included non-guideline papers were relevant to an ED setting (60%) [4, 9, 29, 32, 33, 35, 39, 41, 42, 44–54, 60, 62, 64, 66–70, 75, 76]. There were 21 included non-guideline papers (42%) which were relevant to an ICU setting [28, 30, 34, 36–38, 40, 43, 53, 55–59, 61, 63, 65, 71–74]. The most common patient group was critically ill. Included papers were heterogeneous in terms of airway operator(s) discussed or investigated in the research. Our results include both interventional studies (Table 2) and observational studies (Table 3). Observational studies were the most common (38/50, 76%). Type of VL discussed was also varied, and included Mcgrath MAC, GlideScope, C-MAC (standard balde, D-blade or straight blade), Airtraq, WuScope, AWS, Bullard, Lightward, Karl Storz Video Macintosh Laryngoscope, Stroz C-MAC, V-MAC, flexible fibre optics, hyperangulated VL, standard geometry VL, Olympus, Clarus video system, Truview, Med. Adult type Video Laryngoscope, King Vision, UEScope, Airway Scope, Ambu-Pentax and VividTrac.

There were several different outcomes assessed across the 50 included non-guideline papers. Outcomes included first-pass intubation success, overall intubation success, severe complications of intubation, oesophageal intubation rates, glottic view, frequency of VL use, incidence of difficult intubation and clinical outcomes including hospital length of stay and in-hospital mortality. If outcomes were associated with a statistically significant p-value, this is shown in Tables 2 / 3 for interventional and observational studies respectively.

#### First-pass success

First-pass success was an outcome measure in 30/50 (60%) of included papers [4, 9, 29, 30, 34, 38, 40, 41, 45–52, 54, 56–60, 63–68, 70, 74]. There were 23 papers that directly compared VL and DL in terms of first-pass success. VL was found to be superior to DL in terms of first-pass intubation success in 13/23 (56.5%) of these studies [9, 38, 40, 41, 45, 46, 56, 57, 59, 63, 65, 67, 68]. There was no significant difference between VL and DL in terms of first-pass intubation success in 11/23 (47.8%) of these studies [9, 29, 30, 34, 37, 47–50, 54, 60]. Weng et al. (2020) found that first-pass success was improved with VL compared to DL among non-attending emergency physicians, but not amongst attending emergency physicians and hence is included in both groups here [9].

#### Overall intubation success

Overall intubation success rate was a measured outcome in four of the included studies, with none of these reporting a difference in overall intubation success using VL compared to DL [35, 62, 63, 67].

#### Glottic view

There were 8 included studies that assessed glottic view as an outcome measure. Of these, 7/8 (87.5%) report that VL is associated with improved glottic view compared to DL [31, 34, 42, 44, 54, 65, 66]. Carlson et al. reported that there was no difference in glottic view when using VL compared to DL [47].

#### Oesophageal intubation rate

There were 4 studies that looked at rate of oesophageal intubations, with 100% of these reporting less oesophageal intubations with VL compared to with DL [36, 63, 66, 67].

#### Incidence of difficult intubation

Difficult intubation was an outcome measure in 2 of the included studies, both of which found that the incidence of difficult intubation was less with VL compared to DL [28, 36].

#### Frequency of VL use

There were 10 included studies that report on the frequency of VL use [32, 42, 43, 48, 53, 55, 61, 71–73]. Aziz et al. (2013) reports that the frequency of VL use is growing, as the device is becoming more affordable and more readily available [42]. Results from Brewster et al. (2021), Groombridge (2021) and Hawkins (2021) all suggest that the use of VL has increased with the COVID-19 pandemic [32, 43, 55]. In contrast, Green et al. (2017) and Seisa et al (2018) report that DL remains a more commonly used airway device [53, 71].

#### Complications of intubation

There were eight included studies that discussed complications of intubation using VL. Overall, the results of these studies were varied. Four (50%) of these studies found that there was a difference between VL and DL in terms of complication rates [37, 39, 40, 57], whereas four (50%) did not [28, 29, 34, 35].

#### Clinical outcomes

Clinical outcomes including hospital length of stay and in-hospital mortality rates were measured in only three included studies. There was no statistically significant difference in hospital length of stay, in-hospital mortality or overall mortality found in these studies [29, 31, 34].

## Discussion

This study aimed to provide an up-to-date review of the role of VL in intubation of the critically ill outside the OR. We identified 64 research papers from the past 10 years on the use of VL outside the OR, including 14 clinical practice guidelines.

In terms of first-pass success, some of our included papers (n = 13) showed increased first pass success with VL compared to DL; however, a similar number of papers (n = 11) showed no difference in first-pass success between VL and DL. Weng et al. (2020) found that VL was associated with improved first past success amongst non-attending physicians, but this difference was not seen amongst attending physicians [9]. This suggests that the usefulness of VL in helping to achieve first pass success may be operator dependent. This finding is in keeping with the existing literature, with a review article and meta-analysis from Arulkumaran et al. (2018) demonstrating that first-pass success was increased when VL was used in ICU and amongst novice/trainee clinicians [8]. Furthermore, a review article from Howson et al. (2020) also showed that first-pass success was higher with VL compared to DL in junior operators, but the difference was not seen amongst senior operators [77].

Although some results suggest that VL may improve first-pass intubation success, our results indicate that VL is yet to be shown to improve overall intubation success. We found that VL is likely to improve glottic view, and reduce the incidence of oesophageal intubations; however, the degree to which this view is improved and how this improves clinical outcomes remains unclear. Further research is required to directly determine this.

Most worthy of discussion when reflecting on both first pass success and improved glottic view with VL is the type of VL blade used. There has been a move in recent years towards a Macintosh blade by most VL manufacturers, with an increased focus on the use VL with a hyperangulated blade (HAVL). The papers included in this study have a heterogenous group of video laryngoscopes and blades. HAVL may indeed further improve glottic view, but first-pass success may differ depending on the volume of practice and training in HAVL by the operator [43, 78]. Future research should focus on these outcomes with the use of VL, whilst drawing a distinction between conventional VL blades and HAVL, as well as the use of HAVL in novice versus experienced operator hands.

Our results show that the frequency of VL use outside the OR appears to be increasing in recent years, and particularly through the COVID-19 pandemic. This may be due to VL becoming more affordable and more readily available in both the ICU and ED. Although the exact frequency of VL use outside the OR is uncertain, some studies in our review suggest that the frequency of VL use has increased dramatically during the COVID-19 pandemic [2]. This is likely in response to published clinical practice guidelines on airway management for COVID-19 patients outside the OR unanimously supporting the use of VL in COVID-19 patients, and the perception of safety presented by the increased distance between the airway operator and the patient that VL offers [6]. However, as the COVID-19 pandemic has evolved, we have learnt that the virus is transmitted more by aerosols than my contact, so increased distance between the patient’s airway and the airway operator is perhaps less protective than initially thought. Furthermore, VL is recognised as a powerful training tool, as it allows a supervisor to view to airway in real time and provide real time guidance to a trainee airway operator [9].

Our results were varied in terms of the impact of VL on complications of intubation. Some studies showed that complications such as severe hypoxemia were lower when VL was used, whilst others showed that this complication actually increased with the use of VL. There were 3 studies that looked at clinical outcomes for patients including hospital length of stay and in-hospital mortality rates. There was no statistically significant difference in hospital length of stay, in-hospital mortality or overall mortality found in our included studies. However, the recently published INTUBE study has since also demonstrated an increased likelihood or first-pass success through the use of VL outside the OR [2]. This success correlated with a reduced primary adverse event outcome, being a composite outcome measure that included cardiovascular instability (42.6%), severe hypoxemia (9.3%) and cardiac arrest in 3.1%. Patients in that study with a primary adverse event had an increased hospital mortality (40.7% vs 26.3%) [2]. This should prompt ongoing research to look at specific patient outcomes associated with the use of VL in this patient group.

The primary limitation of this paper is the heterogenous nature of the included papers. Research varied in terms of country of publication, setting, patient group, airway operator(s), study design and type of VL assessed. Specifically, observational studies are analysed alongside RCTs and other interventional studies, which limits the strength of our conclusions. Outcome measures also varied significantly. Most included studies are observational and have a small sample size. In addition, most of the included clinical practice guidelines do not provide a detailed description of their evidence base, or their methodology for guideline development. They also do not describe the use of the same VL manufacturer or blade type. Limited research has been described specifically on the use and benefit of HAVL in this group.

## Conclusion

Ultimately, our results suggest that the use of VL outside the OR has increased in recent years, and particularly through the COVID-19 pandemic. The early use of VL is recommended in most published clinical practice guidelines and is unanimously recommended for management of COVID-19 intubations. It appears that VL is likely to improve glottic view and decrease incidence of oesophageal intubations; however, it remains unclear as to how this contributes to first-pass success. Within the limitations of our research, we found that VL has yet to show significant improvement in overall intubation success or clinical outcomes such as mortality outside the OR. More directed research is required to further characterise the use of VL outside the OR, the type of blade used and the clinical outcomes associated with its use.

## Supporting information

S1 Checklist PRISMA 2009 checklist. (PDF)
